# Supplementary material for: Chromosome structure in Drosophila is determined by boundary pairing not loop extrusion
Source: eLife. 2024 Aug 7;13:RP94070. doi: 10.7554/eLife.94070 (PMC11305675; doi:10.7554/eLife.94070)
Supplement: Supplementary file 2. [file elife-94070-supp2.docx]

Supplemental Data S1. smFISH probes

| **Reagents** | **Source** | **Identifier** |
| --- | --- | --- |
| smFISH probe sets | Biosearch Technologies |  |
| Details below: | | |
| Probes set name | Target | Sequence |
| WK_EGFP | EGFP | cggtgaacagctcctcgc |
|  |  | cagctcgaccaggatggg |
|  |  | ttgtggccgtttacgtcg |
|  |  | tcgccggacacgctgaac |
|  |  | acttcagggtcagcttgc |
|  |  | ttgccggtggtgcagatg |
|  |  | agggtggtcacgagggtg |
|  |  | cactgcacgccgtaggtc |
|  |  | tcggggtagcggctgaag |
|  |  | agtcgtgctgcttcatgt |
|  |  | ggcatggcggacttgaag |
|  |  | tcctggacgtagccttcg |
|  |  | ccgtcgtccttgaagaag |
|  |  | gcgcgggtcttgtagttg |
|  |  | tgtcgccctcgaacttca |
|  |  | cagctcgatgcggttcac |
|  |  | cttgaagtcgatgccctt |
|  |  | caggatgttgccgtcctc |
|  |  | ttgtactccagcttgtgc |
|  |  | agacgttgtggctgttgt |
|  |  | gcttgtcggccatgatat |
|  |  | ccttgatgccgttcttct |
|  |  | ggcggatcttgaagttca |
|  |  | ctgccgtcctcgatgttg |
|  |  | gtagtggtcggcgagctg |
|  |  | gatgggggtgttctgctg |
|  |  | ttgtcgggcagcagcacg |
|  |  | gactgggtgctcaggtag |
|  |  | gttggggtctttgctcag |
|  |  | catgtgatcgcgcttctc |
|  |  | ggtcacgaactccagcag |
|  |  | atgccgagagtgatcccg |
|  |  | tacttgtacagctcgtcc |
| WK_LacZ | LacZ | tttgaggggacgacgacagtat |
|  |  | ataggttacgttggtgtagatg |
|  |  | aaacggcggattgaccgtaatg |
|  |  | ccagctttcatcaacattaaat |
|  |  | tcaaaaataattcgcgtctggc |
|  |  | acagatgaaacgccgagttaac |
|  |  | caaattcagacggcaaacgact |
|  |  | ttttctccggcgcgtaaaaatg |
|  |  | atatcctgatcttccagataac |
|  |  | aacgagacgtcacggaaaatgc |
|  |  | tggaaatcgctgatttgtgtag |
|  |  | aatcatcattaaagcgagtggc |
|  |  | cctgccataaagaaactgttac |
|  |  | atcgataatttcaccgccgaaa |
|  |  | ttttcgacgttcagacgtagtg |
|  |  | cacgatagagattcgggatttc |
|  |  | gatgatgctcgtgacggttaac |
|  |  | taaagttgttctgcttcatcag |
|  |  | atggttcggataatgcgaacag |
|  |  | tttcaatattggcttcatccac |
|  |  | atcatcggtcagacgattcatt |
|  |  | atgatcacactcgggtgattac |
|  |  | ggaaggatcgacagatttgatc |
|  |  | catcgggcaaataatatcggtg |
|  |  | tagcgaaagccattttttgatg |
|  |  | tatttagcgaaaccgccaagac |
|  |  | tgtaaacggggatactgacgaa |
|  |  | tgccgttttcatcatatttaat |
|  |  | aaagaccagaccgttcatacag |
|  |  | aaaactgctgctggtgttttgc |
|  |  | tttgcccggataaacggaactg |
|  |  | cgttatcgctatgacggaacag |
|  |  | tcaatcaactgtttaccttgtg |
|  |  | ctcgatgcaaaaatccatttcg |
|  |  | gttaaattgccaacgcttatta |
|  |  | catctgtgaaagaaagcctgac |
|  |  | cagttgttttttatcgccaatc |
|  |  | cacttacgccaatgtcgttatc |
|  |  | ccggctgataaataaggttttc |
|  |  | catttgaccactaccatcaatc |
|  |  | cacttcaacatcaacggtaatc |
|  |  | ggtcaaaacaggcggcagtaag |
|  |  | tatacatgtctgacaatggcag |
|  |  | gtgtgggccataattcaattcg |
|  |  | gctgatgttgaactggaagtcg |
|  |  | catcagttgctgttgactgtag |
|  |  | tatggaaaccgtcgatattcag |
|  |  | tatttttgacaccagaccaact |
